# Undifferentiated Prehospital Chest Pain: Aetiologies and a Proof-of-Concept Model for Significant Coronary Lesions

**DOI:** 10.3390/jcm15187180

**Published:** 2026-09-16

**Authors:** Sophie Laporal, Olivier Giovannetti, Prabakar Vaittinada Ayar

**Affiliations:** 1 Emergency Department, Orléans University, Orléans University Hospital, 45100 Orléans, France; 2 Laboratoire Interdisciplinaire pour l’Innovation et la Recherche en Santé d’Orléans, Medical School, Orléans University, Orléans University Hospital, 45100 Orléans, France

**Keywords:** chest pain, prehospital care, coronary lesions, predictive score

## Abstract

**Background**: Undifferentiated chest pain is one of the most common reasons for emergency medical service (EMS) activation, yet its aetiological spectrum remains poorly characterised in the prehospital setting. Furthermore, no clinical prediction model has been specifically developed to identify patients at risk of significant coronary lesions using only information available before hospital arrival. This study aimed to describe the aetiologies of undifferentiated prehospital chest pain and develop a proof-of-concept clinical prediction model. **Methods**: We conducted a retrospective, single-centre study including 409 consecutive patients managed by the Orléans Mobile Intensive Care Unit (MICU) for undifferentiated chest pain between January and June 2024. Predictors of clinically significant coronary artery disease identified during in-hospital coronary assessment and management were evaluated using multivariable logistic regression. Model performance was assessed by discrimination and calibration, and internally validated using 1000 bootstrap resamples. **Results**: Cardiological aetiologies accounted for 19% of cases, including 53 patients (13%) meeting the primary outcome of clinically significant coronary artery disease. Four independent predictors were identified: age (OR 6.7–8.9 according to category), male sex (OR 2.2), typical chest pain (OR 6.6), and a positive family history of cardiovascular disease (OR 3.4). These variables were combined to develop the HATS (History, Age, Typical chest pain, Sex) model. The model demonstrated good discrimination (AUC 0.81), and satisfactory internal calibration (Hosmer–Lemeshow *p* = 0.88), with limited optimism after bootstrap validation. **Conclusions**: This study characterises the aetiological spectrum of undifferentiated prehospital chest pain and proposes HATS as an exploratory proof-of-concept prediction model. Prospective multicentre external validation and subsequent assessment of clinical utility are required before any consideration of clinical implementation.

## 1. Introduction

Chest pain is one of the most frequent reasons for calls to emergency medical dispatch centres and for Mobile Intensive Care Unit (MICU) deployment. In France, approximately one third to one half of MICU missions are related to chest pain [1,2,3], and this proportion reached 33% at the Orléans emergency medical dispatch centre in 2024. The immediate priority is to recognise patients with time-sensitive cardiac disease while avoiding unnecessary use of scarce prehospital resources.

This task is particularly difficult when the initial electrocardiogram is non-diagnostic and no clear aetiology can be established before hospital arrival. Clinical presentation is heterogeneous, and access to biomarkers, serial testing, and prolonged observation is limited in the field. Current international guidelines recommend a structured diagnostic approach integrating clinical assessment, ECG interpretation, cardiac biomarkers and, in selected patients, anatomical imaging [4]. Consequently, transport and medicalisation decisions rely largely on symptoms, cardiovascular history, initial examination, and physician judgement [5,6,7,8]. The final diagnostic spectrum of this undifferentiated prehospital population remains incompletely characterised.

Established scores such as TIMI, HEART, and GRACE were developed mainly for in-hospital or emergency-department assessment and may require biomarkers or information not routinely available before hospital arrival [9,10,11]. Although prehospital risk-stratification approaches have been explored [12], evidence remains limited for a model based exclusively on immediately available clinical variables and focused on clinically significant coronary artery disease. A preliminary model could help define candidate predictors and inform the design of prospective multicentre research, but should not be interpreted as ready for clinical use.

The objectives of this study were therefore (1) to describe the final hospital aetiologies among patients managed by a physician-staffed MICU for chest pain without a definitive prehospital diagnosis and (2) to determine whether routinely available clinical variables, rather than novel predictors, could be combined into a parsimonious, internally validated proof-of-concept model for predicting significant coronary lesions in the prehospital setting.

## 2. Materials and Methods

### 2.1. Study Design

This was a retrospective, observational, single-centre study including all patients managed by the Orléans MICU for chest pain without a definitive prehospital aetiological diagnosis. Data were collected over a six-month period from 1 January to 30 June 2024.

### 2.2. Study Population

Inclusion criteria:

All adult patients presenting with chest pain who required MICU intervention and for whom no definitive aetiological diagnosis was established prehospital setting.

Exclusion criteria:

- Patients under 18 years of age.
- Patients with a definitive prehospital aetiological diagnosis.

### 2.3. Data Collection

Data were collected from MICU intervention reports, SAMU dispatch records (e-RS/EXOS), and hospital electronic medical records from Orléans University Hospital (EASILY software, version 8.0, Lyon, France) and Oréliance Clinic (Dopasoins software, version 2.2021.1.12, Artigues-près-Bordeaux, France). All data were anonymised.

Collected variables included demographic data, cardiovascular risk factors [5,6,13,14], clinical presentation, ECG findings, prehospital treatments, transport modality, final hospital diagnosis, coronary angiography findings, and length of stay.

Typical chest pain was defined according to the attending physician’s clinical assessment as constrictive retrosternal chest pain suggestive of myocardial ischaemia, with or without radiation, and compatible with contemporary clinical definitions.

Medicalised transport refers to transport performed by a physician-staffed Mobile Intensive Care Unit (MICU).

### 2.4. Primary Outcome

The primary outcome was clinically significant coronary artery disease identified during in-hospital coronary assessment and management, comprising patients who underwent percutaneous coronary intervention (PCI), patients with coronary artery disease managed medically without PCI, and patients referred for coronary artery bypass grafting (CABG). This outcome was used as the dependent variable in the logistic regression analyses.

The term “significant coronary lesions” is used throughout the manuscript to refer to this study endpoint and should not be interpreted as a purely anatomical definition. Management decisions were made as part of routine clinical care and could reflect coronary anatomy, clinical presentation, comorbidities, and the selected revascularisation strategy.

Coronary CT angiography was routinely available at our institution and was mainly performed in haemodynamically stable patients with atypical chest pain or as part of the outpatient diagnostic work-up when considered appropriate by the treating cardiologist. Its use was not evaluated in the present study, as the objective was not to analyse in-hospital diagnostic strategies.

### 2.5. Statistical Analysis

Data were analysed using STATA 11. Descriptive statistics were expressed as medians or percentages. Univariate analysis was performed using Fisher’s exact test. Variables with *p* < 0.20 in univariate analysis were included in the multivariate logistic regression. Model performance was assessed using receiver operating characteristic (ROC) curve analysis with the area under the curve (AUC), along with the Hosmer–Lemeshow goodness-of-fit test. A predictive score (HATS) was derived from the regression coefficients (β), which were multiplied by 10 to assign a weighted value to each variable included in the model. Internal validation of the final multivariable logistic regression model was performed using bootstrap resampling with 1000 iterations. For each bootstrap sample, the model was refitted and evaluated on both the bootstrap and original datasets in order to estimate optimism. Optimism-corrected estimates of discrimination (area under the receiver operating characteristic curve [AUC]), calibration slope, Brier score, Nagelkerke R^2^, and the observed-to-expected (E/O) ratio were then calculated.

### 2.6. Ethical Approval

This retrospective observational study was conducted in accordance with the Declaration of Helsinki. In accordance with the French data protection authority’s reference methodology (MR-004), the study qualified as non-interventional research and did not require approval from an institutional ethics committee under the French Loi Jardé. The study was approved by the Research Ethics Committee of Orléans University Hospital (CERO 2602-14) and declared to the French Data Protection Authority (CNIL). All data were anonymised before analysis. In accordance with French regulations, all eligible patients received written information describing the study and its objectives and were informed of their right to object to the use of their data for research purposes. Patients were given a one-month period to exercise their right to refuse participation. Patients who objected were excluded from the analysis.

## 3. Results

### 3.1. Study Flow and Population Description

**Figure 1:**
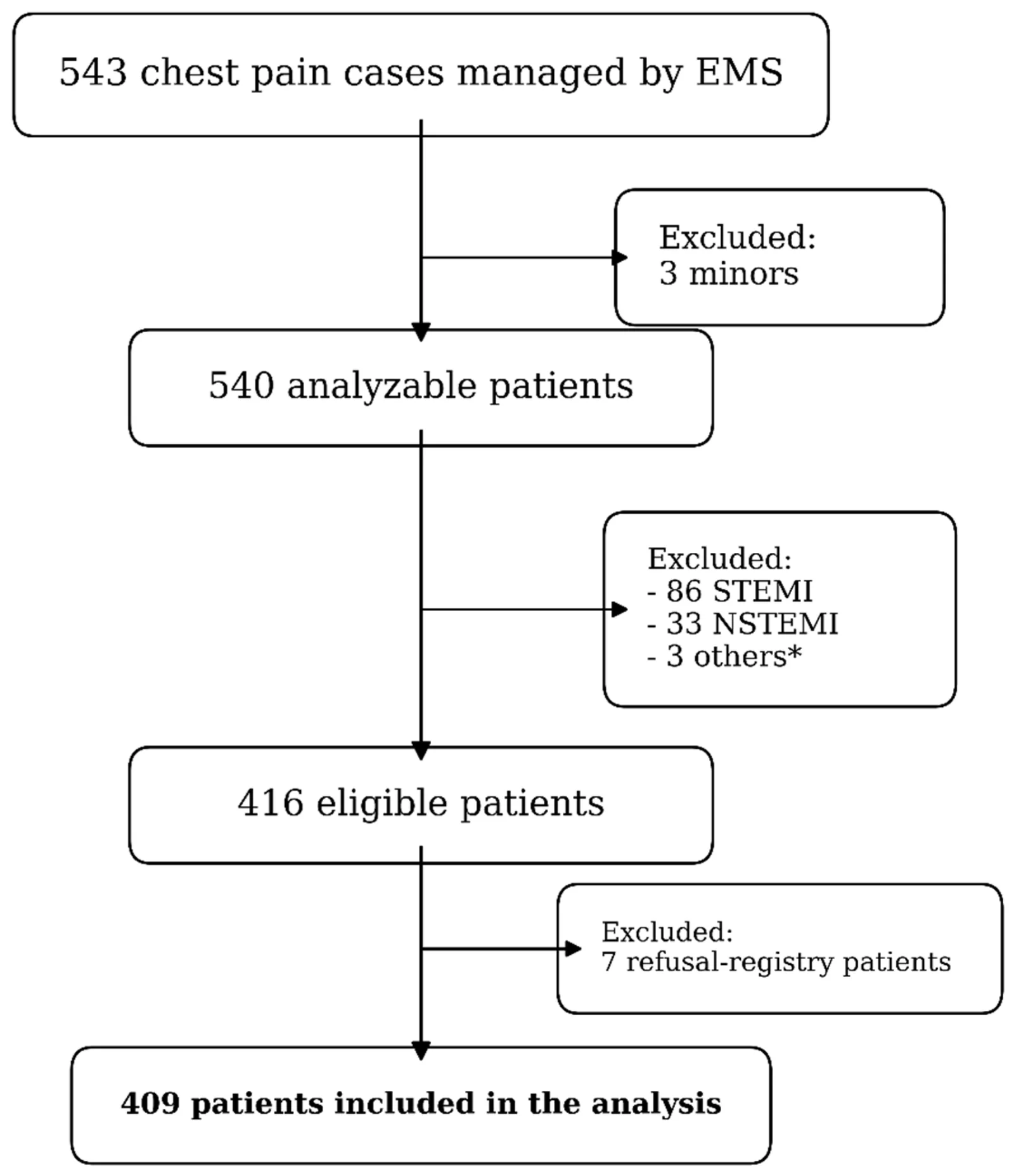
Flow chart detailing patient selection and exclusions. * Detained patient.

Between 1 January and 30 June 2024, a total of 543 patients were managed by the Orléans MICU for chest pain. After exclusion of minors and patients with a clearly established prehospital diagnosis, 409 patients were included in the final analysis (Figure 1).

Among excluded cases, three patients had a final diagnosis established prehospital (ventricular tachycardia in a patient with known ischaemic heart disease, hypertensive acute pulmonary oedema, and clinically suspected pericarditis supported by history and ECG findings).

Baseline demographic, clinical, and prehospital characteristics are summarised in Table 1. A total of 409 patients were included, of whom 53 (13.0%) had significant coronary lesions. The median age was 61 years (IQR, 49–74), and 259 patients (63.3%) were male. Compared with patients without significant coronary lesions, those with coronary lesions were older (median age 69 [IQR, 61–77] vs. 59 [IQR, 48–72] years; *p* < 0.001) and more frequently male (77.4% vs. 61.2%; *p* = 0.034). They also had a higher prevalence of previous ischaemic heart disease (49.1% vs. 28.7%; *p* = 0.004), hypertension (69.8% vs. 42.7%; *p* < 0.001), diabetes (26.4% vs. 14.6%; *p* = 0.043), and a positive family history of cardiovascular disease (37.7% vs. 22.8%; *p* = 0.026). Typical chest pain was markedly more frequent in patients with significant coronary lesions (66.0% vs. 21.1%; *p* < 0.001), as were systolic blood pressure ≥150 mmHg (66.0% vs. 50.0%; *p* = 0.038), a positive nitrate test (55.6% vs. 32.4%; *p* = 0.013), and medicalised transport (49.1% vs. 18.5%; *p* < 0.001). No significant differences were observed regarding dyslipidaemia, smoking status, sweating, malaise, dyspnea, heart failure, or ECG findings.

### 3.2. Final Hospital Diagnoses

**Figure 2:**
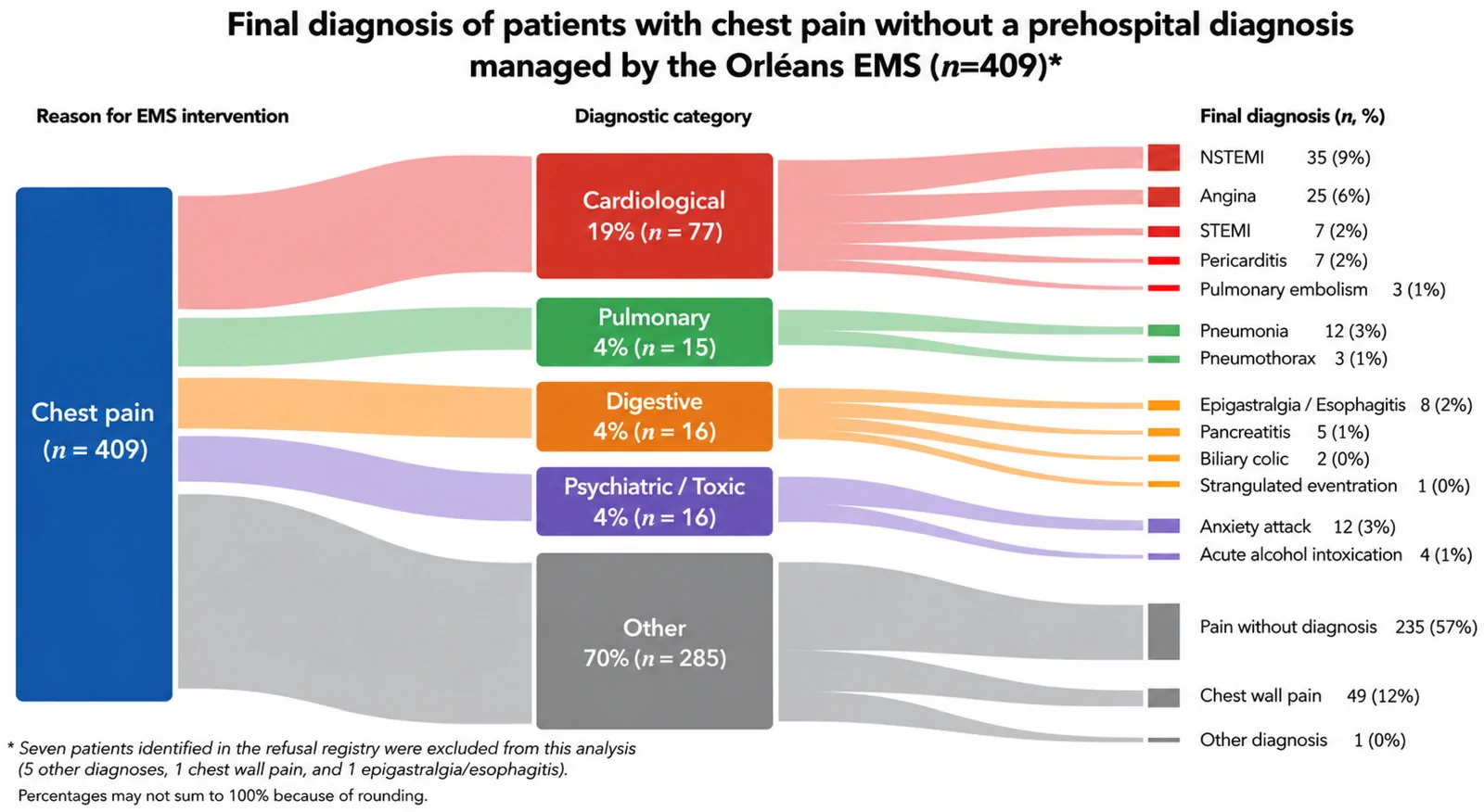
Sankey diagram showing the final diagnoses of patients with chest pain managed by Orléans Mobile Intensive Care Unit without prehospital aetiological diagnosis (*n* = 409). Abbreviations: EMS, emergency medical service.

Final diagnoses at hospital discharge are illustrated in Figure 2. Cardiac aetiologies accounted for 19% of cases, including acute coronary syndromes with or without ST-segment elevation and other ischaemic or rhythm-related conditions. Non-cardiac somatic causes were less frequent and mainly included digestive (4%) and pulmonary (4%) aetiologies. Psychiatric or toxic causes accounted for 4% of diagnoses. Notably, 58% of patients had no definitive somatic diagnosis at hospital discharge. This finding should not be interpreted as evidence of a benign or fully completed diagnostic pathway. Among patients with cardiac aetiologies, several STEMI cases initially presented with non-diagnostic or transient ECG changes, underscoring the dynamic nature of acute coronary syndromes and the limitations of a single prehospital assessment.

### 3.3. Significant Coronary Lesions

Overall, 53 patients (13%) met the primary outcome of clinically significant coronary artery disease. Among them, 45 underwent percutaneous coronary intervention (PCI), 4 had coronary artery disease managed medically without PCI, and 4 were referred for coronary artery bypass grafting (CABG). Overall, 74 patients (18%) underwent invasive coronary angiography during hospital management (see Supplementary Table S1). These patients represented nearly 70% of those ultimately diagnosed with a cardiac aetiology (*n* = 77; 19%). Patients with significant coronary lesions were more frequently male, older, and more likely to present with typical chest pain, elevated systolic blood pressure, positive nitrate testing, and a positive family history of cardiovascular disease. They were also significantly more likely to be transported with physician-staffed medicalisation.

### 3.4. Univariate and Multivariable Analyses

Variables associated with significant coronary lesions in univariate analysis (Table 1) at a threshold of *p* < 0.20 were included in the multivariate model. Multivariable logistic regression identified four independent predictors of significant coronary lesions: age, male sex, typical chest pain, and positive cardiovascular family history (Table 2). Increasing age was associated with a marked and progressive rise in risk, with odds ratios exceeding 8 beyond the age of 64 years.

### 3.5. Model Performance and HATS Score

**Figure 3:**
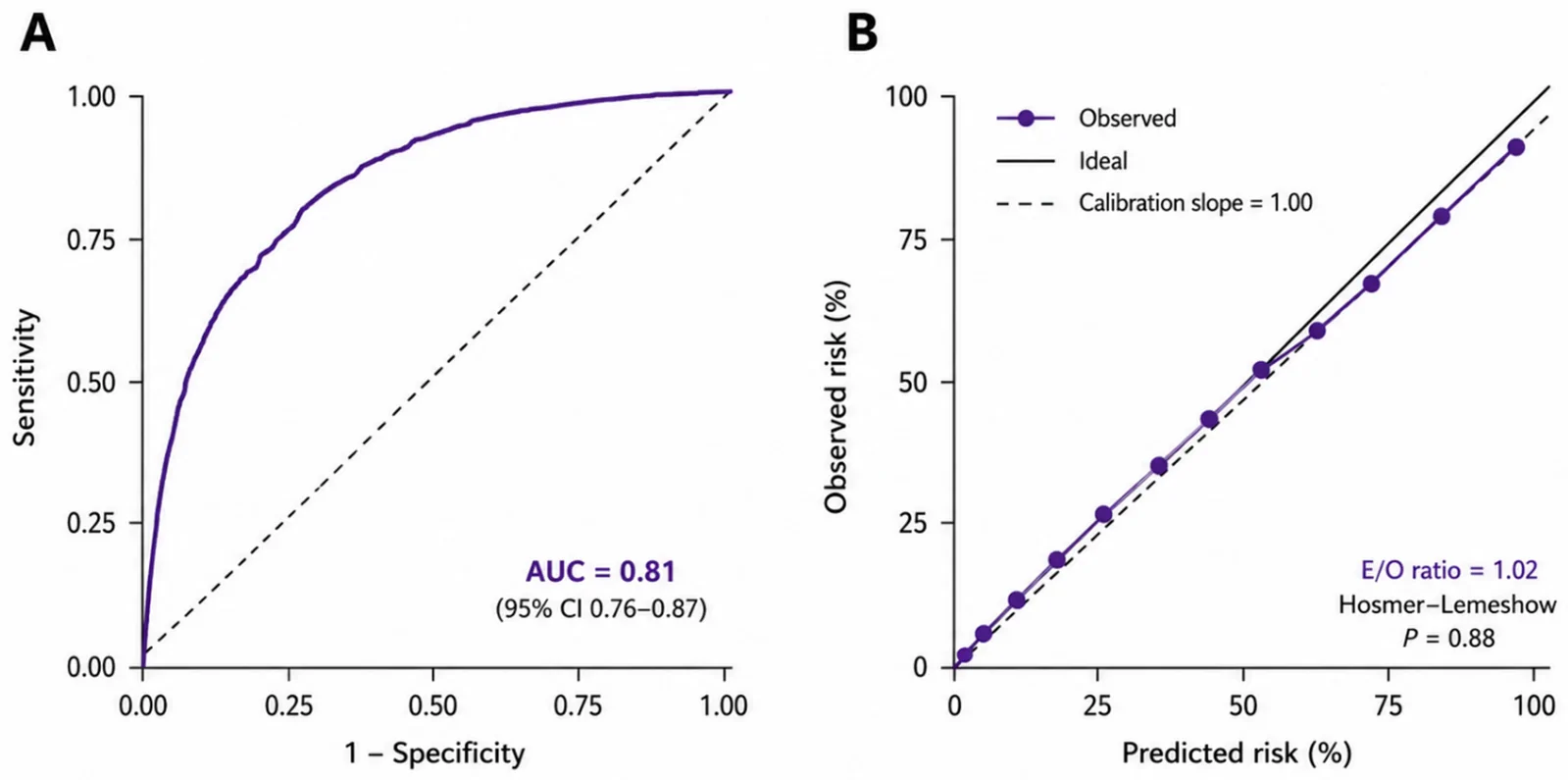
Performance of the HATS score for predicting clinically significant coronary artery disease. (**A**) Receiver operating characteristic (ROC) curve showing the discrimination of the HATS score (AUC 0.81, 95% CI 0.76–0.87). (**B**) Calibration plot comparing predicted and observed probabilities of clinically significant coronary artery disease (Hosmer–Lemeshow *p* = 0.88; observed-to-expected ratio = 1.02). Abbreviations: AUC, area under the receiver operating characteristic curve; CI, confidence interval; E/O, observed-to-expected ratio.

The prediction model demonstrated good discrimination, with an area under the ROC curve of 0.81 (Figure 3A), and satisfactory internal calibration, as indicated by a non-significant Hosmer–Lemeshow test (*p* = 0.88) and close agreement between predicted and observed events across deciles of predicted risk (Figure 3B).

Based on regression coefficients, the HATS score—History, Age, Typical chest pain and Sex—was constructed (Table 3).

Internal validation using 1000 bootstrap resamples demonstrated limited optimism (Table 4). The apparent AUC was 0.826 and the optimism-corrected AUC was 0.810. The apparent Brier score was 0.085 and the optimism-corrected Brier score was 0.091. The apparent Nagelkerke R^2^ was 0.305 and the optimism-corrected Nagelkerke R^2^ was 0.232. The optimism-corrected calibration slope was 0.91, and the E/O ratio was close to 1.0, indicating good discrimination and satisfactory calibration of the HATS model.

## 4. Discussion

In this retrospective single-centre cohort of 409 patients with undifferentiated prehospital chest pain, the main contribution was twofold. First, the study characterised the final diagnostic spectrum of a population in whom a physician-staffed prehospital assessment had not established an aetiology. Second, it identified four readily available variables associated with significant coronary lesions and combined them in an internally validated exploratory model. These findings are hypothesis-generating and should be viewed as a basis for further multicentre investigation rather than evidence for immediate implementation of a new decision rule.

### 4.1. Etiological Spectrum of Chest Pain

Despite prehospital medical assessment, fewer than one quarter of patients received a definitive etiological diagnosis before hospital admission, highlighting the intrinsic difficulty of chest pain evaluation in the prehospital setting. Even after the in-hospital work-up, nearly 60% of patients remained without a clearly identified somatic cause. Similar findings have been reported in contemporary ambulance and emergency department cohorts, where a substantial proportion of patients presenting with chest pain ultimately receive a diagnosis of non-specific chest pain or no definitive somatic diagnosis [15,16,17,18]. In the present study, the absence of a definitive somatic diagnosis should not necessarily imply that symptoms are benign, as non-cardiac chest pain frequently required further outpatient evaluation and management [19].

Nevertheless, nearly one fifth of patients had a final cardiac diagnosis, and 13% met the primary outcome of clinically significant coronary artery disease. This proportion is in line with international reports describing coronary causes in 15–25% of chest pain presentations, despite the selected nature of our population, which had already been deemed at higher risk by the dispatch physician [16,20].

### 4.2. Medicalisation and Clinical Judgement

Approximately 22% of patients were transported with physician-staffed medicalisation. Importantly, half of the patients with significant coronary lesions were medicalised, compared with less than 20% of those without such lesions. This observation suggests that prehospital clinical judgement is already effective at identifying higher-risk patients, even in the absence of a formal diagnosis [21].

Multivariate analysis focusing on medicalisation confirmed that this decision was driven primarily by objective clinical features such as typical chest pain, diaphoresis, and known ischaemic heart disease, rather than by physician-related factors. However, the fact that more than half of patients with significant coronary lesions were not medicalised underscores the potential value of further research in prehospital risk stratification approaches [12].

### 4.3. Predictors of Significant Coronary Lesions

Four independent predictors emerged from the multivariate analysis: age, male sex, family history of cardiovascular disease, and typical chest pain. These factors are well-established determinants of coronary artery disease and are incorporated into many in-hospital risk stratification scores [9,10,20]. Previous studies have also shown that combining cardiovascular risk factors with ECG findings may improve early risk stratification in patients presenting with chest pain [22]. Their identification in a purely prehospital context reinforces the central role of clinical assessment when paraclinical resources are limited.

Age was the strongest predictor, with a sharp increase in risk beyond the age of 50 years and particularly after 64 years. Male sex doubled the risk, reflecting known sex-related differences in coronary disease epidemiology [23]. Family history was associated with a threefold increase in risk, consistent with its recognised role as a major cardiovascular risk factor [24]. Typical chest pain remained a strong predictor, although one third of patients with significant coronary lesions did not report typical symptoms, highlighting the well-known variability of acute coronary syndrome presentation [20].

Traditional cardiovascular risk factors such as hypertension, diabetes, and smoking did not remain in the final multivariate model. This likely reflects collinearity with age and sex rather than the absence of a true association with coronary artery disease [25].

### 4.4. Exploratory Prediction Model and Research Implications

The HATS model was derived from four variables available during the initial prehospital assessment. Its apparent discrimination, calibration, and bootstrap-corrected performance support the internal coherence of the model and the feasibility of developing a prediction model for clinically significant coronary artery disease using clinical variables available during the initial prehospital assessment. This differs from widely used in-hospital scores such as GRACE, TIMI, and HEART, which were developed for other populations and purposes [9,10,11]. Current ESC Guidelines emphasise that definitive diagnosis relies on serial ECGs, high-sensitivity troponins and, when appropriate, coronary imaging, highlighting that HATS should be viewed as an exploratory approach to prehospital risk stratification rather than as a diagnostic algorithm or clinical decision rule [26]. The HATS model is not intended to replace guideline-directed diagnostic pathways but rather to explore whether routinely available clinical variables can support prehospital risk stratification [4].

Importantly, the purpose of the HATS model is not to replace physician judgement or to identify novel predictors of coronary artery disease. Rather, it seeks to formalise routinely available bedside clinical information into a simple, standardised prediction model that can be objectively evaluated and externally validated. The proof-of-concept demonstrated by the present study therefore lies in the feasibility of constructing such a model using only variables available during the initial prehospital assessment.

However, the observed association between higher HATS values and clinically significant coronary artery disease was estimated in the same cohort used to develop the model. The score should therefore be considered a proof-of-concept rather than a clinical decision rule. The present study was designed to derive and internally validate a prediction model, not to determine whether its use improves physician judgement or patient management. Demonstrating incremental value over routine clinical assessment requires prospective implementation studies and necessarily represents a subsequent stage following model derivation and external validation [12].

### 4.5. Strengths and Limitations

The strengths of this study include detailed prehospital clinical data and in-hospital follow-up, which allowed final diagnoses and coronary management to be ascertained. Several limitations nevertheless constrain interpretation. The retrospective design may have introduced missingness and measurement error, particularly for subjective features such as pain characteristics. Furthermore, diagnoses were extracted from the final hospital discharge records and were not independently adjudicated. No systematic post-discharge follow-up was available, which may have resulted in under-recognition of diagnoses established after hospital discharge. The single-centre, physician-staffed French system and the restriction to patients selected for MICU assessment limit transportability to other emergency medical services and may have enriched the cohort for higher-risk presentations. The simplified definition of family history of cardiovascular disease may have caused misclassification. The primary outcome was treatment-informed rather than based on a purely anatomical definition and may therefore partly reflect local clinical management decisions. The modest number of outcome events and absence of an independent validation cohort mean that performance may still be optimistic despite bootstrap correction. In addition, two components of the HATS model may have limited reproducibility in routine clinical practice. Family history was assessed using a simplified definition and may therefore have been subject to misclassification. Similarly, the characterisation of typical chest pain relies partly on clinical judgement and may be subject to interobserver variability. Because both variables contribute directly to the HATS model, their reproducibility should be specifically evaluated in future prospective external validation studies. Finally, no long-term follow-up was available to assess major adverse cardiovascular events after discharge. Accordingly, HATS remains an exploratory model requiring prospective external validation and clinical-utility assessment before any consideration of routine use [20].

## 5. Conclusions

Among patients with undifferentiated chest pain assessed by a physician-staffed MICU, cardiac diagnoses remained clinically important, and 13% met the primary outcome of clinically significant coronary artery disease. Age, male sex, typical chest pain, and a positive family history of cardiovascular disease formed an internally validated exploratory model with good apparent performance. HATS should be regarded as a proof-of-concept model that generates hypotheses for prospective multicentre validation and subsequent clinical-utility assessment, rather than as a tool for current clinical decision-making.

## Figures and Tables

**Table 1:** Baseline demographic, clinical and prehospital characteristics.

Characteristics	Total Population (*n* = 409)	Significant Coronary Lesions (*n* = 53)	No Significant Coronary Lesions (*n* = 356)	*p*
**Demographics**				
Age, years	61 (49–74)	69 (61–77)	59 (48–72)	<0.001
Sex				
Male	259 (63.3%)	41 (77.4%)	218 (61.2%)	0.034
Female	150 (36.7%)	12 (22.6%)	138 (38.8%)	
Age				
<51 years	115 (28.1%)	3 (5.7%)	112 (31.5%)	0.001
52–63 years	108 (26.4%)	17 (32.1%)	91 (25.6%)	
64–74 years	95 (23.2%)	17 (32.1%)	78 (21.9%)	
≥75 years	91 (22.2%)	16 (30.2%)	75 (21.1%)	
BMI				
Normal <25	120 (30.8%)	20 (37.7%)	100 (29.7%)	0.046
Overweight 25–30	156 (40.0%)	13 (24.5%)	143 (42.4%)	
Obesity ≥30	114 (29.2%)	20 (37.7%)	94 (27.9%)	
**Past medical history**				
Ischaemic heart disease	128 (31.3%)	26 (49.1%)	102 (28.7%)	0.004
Hypertension	189 (46.2%)	37 (69.8%)	152 (42.7%)	<0.001
Diabetes	66 (16.1%)	14 (26.4%)	52 (14.6%)	0.043
Dyslipidaemia	178 (43.5%)	28 (52.8%)	150 (42.1%)	0.181
Smoking				
Non-smoker	207 (50.6%)	22 (41.5%)	185 (52.0%)	0.175
Current smoker	110 (26.9%)	14 (26.4%)	96 (27.0%)	
Former smoker	92 (22.5%)	17 (32.1%)	75 (21.1%)	
Family history of cardiovascular disease	101 (24.7%)	20 (37.7%)	81 (22.8%)	0.026
**Clinical and prehospital characteristics**				
SBP ≥ 150 mmHg	213 (52.1%)	35 (66.0%)	178 (50.0%)	0.038
Typical chest pain	110 (26.9%)	35 (66.0%)	75 (21.1%)	<0.001
Sweating	24 (5.9%)	6 (11.3%)	18 (5.1%)	0.107
Malaise	39 (9.5%)	4 (7.5%)	35 (9.8%)	0.803
Heart failure	13 (3.2%)	3 (5.7%)	10 (2.8%)	0.23
Dyspnea	26 (6.4%)	5 (9.4%)	21 (5.9%)	0.36
Positive nitrate test (among tested)	68 (37.0%)	20 (55.6%)	48 (32.4%)	0.013
Normal ECG	229 (56.0%)	26 (49.1%)	203 (57.0%)	0.301
ECG with known repolarisation abnormalities	34 (8.3%)	6 (11.3%)	28 (7.9%)	0.421
ECG with non-significant repolarisation abnormalities	80 (19.6%)	11 (20.8%)	69 (19.4%)	0.853
Medicalised transport	92 (22.5%)	26 (49.1%)	66 (18.5%)	<0.001

Values are *n* (%). Percentages are calculated on available data. BMI percentages use the non-missing BMI denominator (*n* = 390). Positive nitrate test percentages use patients with a nitrate test documented (*n* = 184). *p* values were recalculated from updated aggregated counts using Fisher exact tests for binary variables and chi-square tests for multi-category variables. Abbreviations: BMI, body mass index; SBP, systolic blood pressure; ECG, electrocardiogram.

**Table 2:** Multivariable logistic regression analysis.

Predictors		Coefficient β	OR	95% CI	*p*
Family History of cardiovascular disease		1.24	3.44	1.61–6.60	0.001
Age	52–63 years	1.9	6.67	1.77–25.07	0.005
	64–74 years	2.16	8.64	2.24–33.4	0.002
	≥75 years	2.18	8.87	2.25–35	0.002
Typical chest pain		1.88	6.56	3.45–12.84	<0.001
Male Sex		0.79	2.2	1.02–4.55	0.04

OR = Odd Ratio − CI = confidence interval.

**Table 3:** HATS Score.

Predictors		Points
Male Sex		8
Age	52–63 years	19
	≥64 years	22
Family History of cardiovascular disease		12
Typical chest pain		19

**Table 4:** Internal validation of the HATS prediction model.

Metric	Apparent Model	Optimism-Corrected Model	Comment
Sample size	409	—	Adult patients after excluding refusal registry
Events	53 (12.9%)	—	Clinically significant coronary artery disease (primary outcome)
AUC	0.826	0.810	Optimism-corrected after bootstrap
AUC optimism	—	0.016	Mean bootstrap optimism from previous run
Brier score	0.085	0.091	Lower values indicate better overall accuracy
Calibration slope	1.00	0.91	Shrinkage factor
E/O ratio	1.00	1.01	Observed/expected events
Nagelkerke R^2^	0.305	0.232	Bootstrap optimism-corrected

Internal validation was performed using bootstrap resampling (1000 iterations). Abbreviations: AUC, area under the receiver operating characteristic curve; E/O, observed-to-expected ratio; R^2^, coefficient of determination.

## Data Availability

All study data are available upon reasonable request from the corresponding author.

## Supplementary Materials

The following supporting information can be downloaded at: https://www.mdpi.com/article/10.3390/jcm15187180/s1, Table S1: Coronary anatomical investigations performed during hospital management (N = 409).
