# Supplementary material for: Undifferentiated Prehospital Chest Pain: Aetiologies and a Proof-of-Concept Model for Significant Coronary Lesions
Source: J Clin Med. 2026 Sep 16;15(18):7180. doi: 10.3390/jcm15187180 (PMC13607085; doi:10.3390/jcm15187180)
Supplement: Supplementary file 1 [file jcm-15-07180-s001.zip › jcm-4488271-supplementary.pdf]

Supplementary

Investigation	n (%)
**Invasive coronary angiography**	**74 (18.1)**
**Percutaneous coronary intervention (PCI)**	**45 (11.0)**
**Obstructive coronary artery disease managed medically (no PCI)**	**4 (1.0)**
**Referral for coronary artery bypass grafting (CABG)**	**4 (1.0)**
**No obstructive coronary artery disease (normal or non-significant coronary lesions)**	**21 (5.1)**
**Coronary CT angiography**	**41 (10.0)**
**Performed during hospitalisation**	**6 (1.5)**
**Scheduled or performed as an outpatient investigation**	**35 (8.6)**

Table S1. Coronary anatomical investigations performed during hospital management (N = 409)
